# Wood Density and Moisture Content Estimation by Drilling Chips Extraction Technique

**DOI:** 10.3390/ma13071699

**Published:** 2020-04-05

**Authors:** Roberto D. Martínez, José-Antonio Balmori, Daniel F. Llana, Ignacio Bobadilla

**Affiliations:** 1 Timber Structures and Wood Technology Research Group, Universidad de Valladolid, 47014 Valladolid, Spain; balmori@arq.uva.es; 2 Timber Construction Research Group, UPM, 28040 Madrid, Spain; danielfllana@gmail.com (D.F.L.); i.bobadilla@upm.es (I.B.); 3 Timber Engineering Research Group, H91HX31 NUI Galway, Ireland; 4 Department of Forest and Environmental Engineering and Management, MONTES (School of Forest Engineering and Natural Resources), Universidad Politécnica de Madrid, 28040 Madrid, Spain

**Keywords:** density estimation, MC adjustment, drilling residue, non-destructive testing, wood extractor, timber structures assessment

## Abstract

The novelty of this study is the development of an accurate wood moisture content (MC) estimation method based on a relatively brand-new, non-destructive testing technique (drilling chips extraction). The method is especially important in the assessment of existing timber structures, where non-destructive testing (NDT) results are affected by wood MC and should be adjusted to a reference MC, usually 12%. In the assessment of timber structures, it is not possible to determine MC by oven drying method and this should be estimated. Electrical resistance and capacitance are the conventional methods used for MC estimation. This research work aims to present an accurate MC estimation method based on the drilling chips extraction technique. For that, 99 specimens (90 × 65 × 38 mm^3^) from three softwood and hardwood species covering a wide range of densities (from 355 to 978 kg m^−3^) were tested after conditioning at five different MCs (5%, 10%, 15%, 20%, 25%). The Wood Extractor device based on the drilling chips extraction technique was used. The mass of the chips collected (drilling residue) from each drill was recorded. The results show that the MC of the chips extracted was statistically significantly different than the MC of the specimen and cannot be directly used as MC determination. However, the chips MC can be used as an estimator of specimen MC with high determination coefficients (R^2^ from 71% to 86%). As the main result, models to estimate density directly adjusted to a reference 12% MC from the wet and dry mass of chips extracted were developed with an R^2^ of 98%. In sum, the drilling chips extractor is a dependable and straightforward method to estimate MC and density from only one measurement. Density adjusted to a reference 12% MC can be directly estimated from a single model.

## 1. Introduction

Non-destructive testing (NDT) is widely used in the assessment of existing timber structures for wood damage evaluation (internal defects and biological degradation), and for the estimation of the wood mechanical properties [1,2] due to the fact that these techniques are easy to use, dependable and accurate [3,4,5,6].

In order to increase the assessment accuracy of timber structures for its characterization, it is a common practice to combine several different NDT methods [7,8,9]. Wood density estimation from NDT methods is usually the most important result because it is well correlated with wood’s physical and mechanical properties [10,11,12]. Probing, coring and drilling are NDT techniques commonly used in the assessment of existing timber structures to estimate density. The most common probing techniques are penetration depth and pull-out resistance. These are inexpensive and easy to use methods for density estimation, but the determination coefficients (R^2^) are lower than 70% [13,14,15]. The coring technique was adapted from the increment borer used in standing trees to the assessment of timber structures. It is also inexpensive, easy to use and an R^2^ of up to 89% for density estimation was achieved [15,16], but the damage is more significant than probing or drilling techniques. Resistograph devices mainly conduct drilling technique. These are expensive tools, and complicated models are developed for density estimation with an R^2^ up to 90% [17,18]. However, the estimation of density on existing structures is not always successful using this technique [19]. Drilling chips’ extraction technique was recently used for wood density estimation with high accuracy, with R^2^ values achieved of 84% for softwood species [15,20,21] and an R^2^ of 97% for softwood and hardwood species [22]. Furthermore, the dynamic modulus of elasticity (Edyn) can be calculated from the estimated density and other NDT results, such as wave or resonance velocity [23,24,25].

NDT results are influenced by different factors related to internal wood structure (grain angle), test condition (device, sensor positioning), or environmental conditions (moisture content (MC), temperature) [26]. The influence of MC over NDT results is the most important, and several research and standardization works have proposed different MC adjustment factors [27]. Most of the research works related to the MC influence on NDT results focused on acoustic and resonance techniques [28,29,30,31,32]. Most of them found a more substantial MC influence below than above the fiber saturation point (FSP) [33,34,35,36,37]. Fewer research works dealing with MC influence on NDT-punctual techniques (probing, drilling, and coring) were found [38,39,40]. A more substantial MC influence below FSP was also reported for these techniques [41,42,43,44]. These techniques are usually used in the assessment of timber structures where the MC is below FSP.

In the assessment of timber structures, several acoustic and punctual NDT techniques are used, and MC should adjust the results. However, the MC cannot be determined by the drying oven method and an accurate method to estimate the wood MC in situ is needed. Several devices are available to estimate MC based on electrical resistance and capacitance methods [45,46]. The capacitance method is commonly used in sawmill lines for new timber and can estimate MC only a few mm inside the timber. It is not a suitable method in the assessment of timber structures as many pieces are large cross-section, and the MC estimation is only superficial. In the case of electrical resistance method, most devices can be connected to long pins that can be introduced further inside the timber for MC estimation. It is not accurate for the estimation of the entire range of MC, as the relationship between the electrical properties and the MC suffers considerable variation above FSP.

The methods showed above imply the use of two different equipment, one for the estimation of the density and another for the estimation of MC. The proposal of this work is to estimate everything with a single device and a single measurement.

The specific objective of the present study is to show a brand-new NDT method to accurately estimate wood density directly adjusted to a reference value of 12% MC based on the drilling chips’ extraction technique allowing the fast and accurate assessment of timber structures. Furthermore, MC would be estimated from the same measurement.

## 2. Materials and Methods

### 2.1. Wood Specimens

A total of 99 small clear specimens from three softwood and hardwood species, covering a wide range of density (from 355 to 978 kg m^−3^), were used. One species of very low density (Western red cedar), one of medium density (Salzmann pine), and one of very high density (Missanda) were selected. From each species, 33 specimens were obtained from the same piece of heartwood, avoiding areas with defects such as fissures, knots and resin pockets (Table 1). The specimens were tested at five different MC in the range from 5% to 25%.

The relationship between the density of the specimen and the mass of the chips extracted during drilling is linear [21,22], as well as between the MC of the specimen and the MC of the chips removed [20]. In this way, the line is defined with the ends, and the linearity is checked with a species in the centre.

In order to have a quicker wetting or drying velocity, specimens were obtained in such a way that the longitudinal direction with respect to the rings was the shortest dimension. In that way, the exchange of water vapour between the outside and the inside of the specimen was maximised.

After conditioning to the desired MC, specimens’ sizes with a resolution of 0.01 mm and mass with a resolution of 0.01 g were recorded. Density was calculated according to Equation (1); where ρ is the density in kg m^−3^, m is the mass in kg and v is the volume in m^3^.

ρ = m/v(1)

### 2.2. Drilling Residue Collection Device

**Figure 1:**
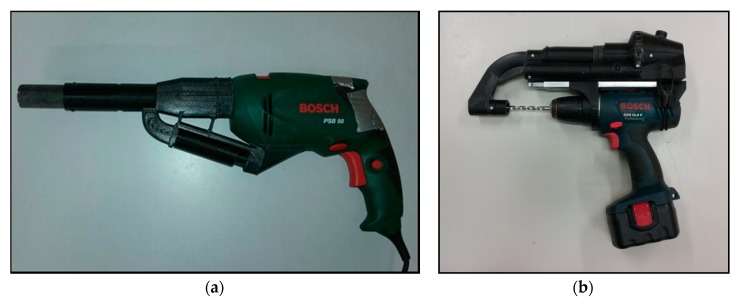
Wood extractor device, (**a**) 8 mm diameter drill bit configuration; (**b**) 7 mm diameter drill bit configuration.

The wood extractor device developed by Martinez and Bobadilla [47] has been used to estimate the MC and the density of wood at 12% MC, Figure 1. This device was designed to be coupled to a commercial power drill to collect all the waste that is produced during drilling in a single-use paper bag filter [20,21]. This technique involves setting drill diameter and depth, giving a known removed volume of wood. After drilling and the collection of residues in the filter, the sample is weighed to estimate wood density [20,21].

In this study, two different configurations have been used. The first was an 8 mm diameter drill bit and a depth of 47.7 mm, giving a 2.4 cm^3^ volume of chips removed with a one-use filter mass of 0.27 g The operation of this configuration is based on harnessing the movement of air produced by the turbine of a conventional drill to suck up the chips produced by drilling a hole and encapsulating them in the one-use filter. The second was a 7 mm diameter drill bit and a depth of 65 mm, giving a 2.5 cm^3^ volume of chips removed with a one-use filter mass of 0.40 g. The operation of this configuration is based on an external vacuum turbine to suck up the chips produced by drilling a hole and encapsulating them in the one-use filter [48].

### 2.3. Humidification Chamber

**Figure 2:**
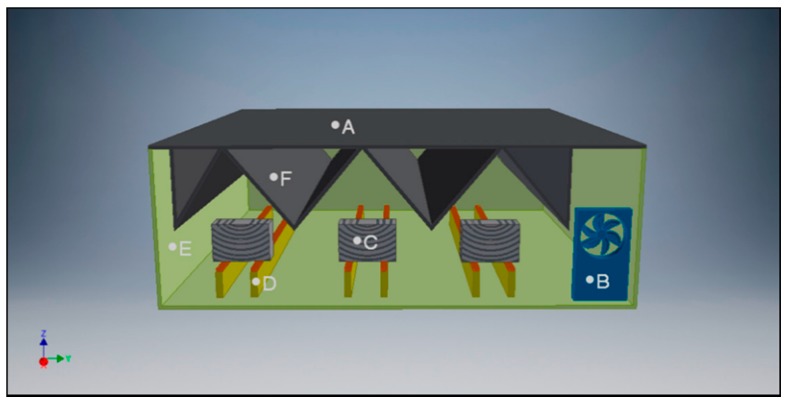
Scheme of the humidification chamber. A: lid, B: fan, C: wooden specimen, D: support, E: cuvette and F: baffle.

The humidification chamber consists of a plastic cuvette with a sealed lid inside where there are three pairs of supports for specimen placing, Figure 2. These supports avoid the contact between the specimens and the water at the bottom of the cuvette. On top of these supports, there is an alveolar plastic coating whose function is to minimize the contact surface and maximize the vapour exchange surface of the specimen.

The lid has baffles preventing the fall of condensation water on the specimens and redirecting it to the spaces between specimens. In addition, these baffles are positioned in such a way that, when opening the lid, the condensation water is discharged away from the specimens. This is an essential feature, since inside the chamber the air is saturated, and any temperature decrease would cause the dew point to be reached. In any case, the increased wood moisture through direct contact with water is not allowed.

The humidification chamber has a fan inserted at a small angle on the water surface to speed up the conditioning process and maintain stable conditions inside the chamber. This fan allows the faster evaporation of the water while homogenizing the relative humidity of the air inside the chamber. The airflow creates a localized atmospheric pressure drop, which causes a decrease in water vapour pressure which results in a higher evaporation compared to static conditions. The fan action is controlled by a timer, established for the present study in 15 min working every three hours. Finally, the humidification chamber is located in the laboratory at 20 °C.

### 2.4. Humidification Chamber Use Protocol

The bottom of the cuvette was filled with water until it reaches 2 or 3 cm depth, avoiding contact with the alveolar covering of the supports. The specimens were placed centred on each pair of supports, and the cuvette was closed with the lid. The mass of each specimen was daily recorded. When the specimens reached the mass corresponding to the target MC, they were removed from the humidification chamber and placed in a zip bag (sealed) to stabilize their humidity. After one week, each specimen mass was re-recorded, and if its mass value didn’t vary, the specimen was considered as stabilized. If its mass value varied, the specimen was reintroduced into the humidification chamber again, and the process was repeated.

### 2.5. Reach the Target Moisture Content (MCt)

For each species, 33 specimens were placed in a humidification chamber to be stabilized at 20 ± 2 °C and 55% ± 5% RH (10% equilibrium MC). Once stabilized, three control specimens were separated, and the average of their moisture content (MC_S_) was determined according to Standard EN: 13183–1: 2002 [49]. This MCs was used to calculate the theoretical wet mass that the other specimens must reach employing Equation (2), where m_MCt_ is the mass of the specimen at the target MCt; mw is the wet mass of the specimen; MCs is the average moisture content of the three control specimens; MCt is the specimen moisture content target.

(2) $$m_{\mathrm{MCt}}=\frac{m_{w}\left(100+\mathrm{MCt}\right)}{{\mathrm{MC}}_{s}+100}$$

The wet mass (m_w_) of remaining specimens was recorded and the specimens were organized into five groups of six specimens each. A moisture content target (MCt) was assigned to each group. Therefore, the following groups were formed: Group 1 (MCt = 5%); Group 2 (MCt = 10%); Group 3 (MCt = 15%); Group 4 (MCt = 20%); and Group 5 (MCt = 25%).

Because it is unlikely to obtain the exact m_MCt_ for each specimen, it was considered that the MCt was achieved when the mw of the specimen was such that the MC was in the range of MCt ± 1%. Therefore, when mw ϵ (m_MCt−1_; m_MCt+1_), see Table 2.

When calculated (m_MCt−1_; m_MCt+1_) for each specimen, they were separated into three batches: the specimens that needed to lose mass to reach their MCt, belonging to Group 1; the specimens that were already in their MCt, belonging to Group 2; and the specimens that needed to gain mass to reach their MCt, belonging to Groups 3, 4 and 5.

To achieve the target mass of the Group 1, these specimens were introduced in an oven at 70 °C, checking their mass every two hours until they reached their m_MCt_.

In the case of Group 3, 4 and 5, these specimens were introduced into the humidification chamber until they acquired the corresponding m_MCt_.

Once the specimens reached MCt, they were stored in zip bags for stabilization. After a week, their m_MCt_ was checked, they were considered stabilized, and their test was carried out.

### 2.6. Drill Residue Extraction

**Figure 3:**
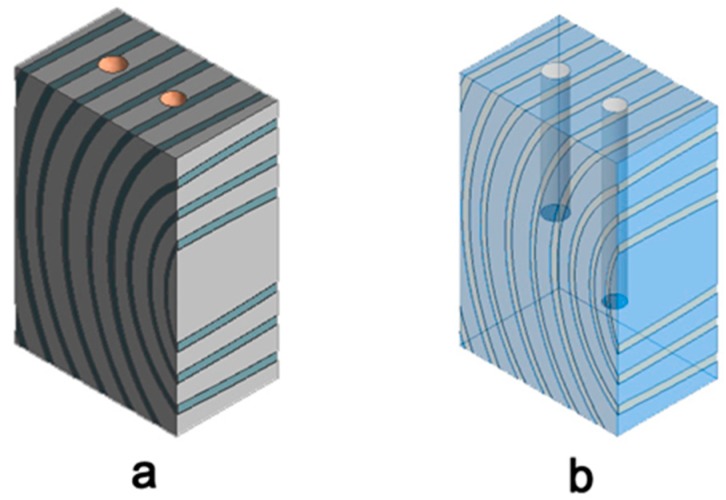
Specimen (**a**) Location of the different tests; (**b**) Projection of the holes inside the specimen, the difference in the depth of the drills made can be seen in the image.

Two samples were taken per specimen, Figure 3, one with the 8 mm diameter drill bit extracting 2.4 cm^3^ and the other with the 7 mm diameter drill bit 2.5 cm^3^. The mass of the extracted chips including the filter (dr_w_), and then the dry mass of the filled filter (dr_0_) was recorded, and its MC was determined by the drying oven method according to Standard EN: 13183-1: 2002 [49].

According to previous studies, no statistically significant differences in the density estimation according to the test direction (radial or tangential with respect to the ring) were found [13,15]. Therefore, there is not any direction limitation in the test of the specimens.

### 2.7. Corrected Density at 12% MC

To obtain the density of the specimen at 12% MC, Equation (3) proposed by EN 384:2016+A1:2019 [50] was applied, where “ρ12” is the density of the piece to be estimated, corrected at 12% MC (kg m^−3^); “ρ_MC_” is the density of the piece to their MC (kg m^−3^) and MC is the moisture content of the piece (%).

(3) $$\rho_{12}=\rho_{\mathrm{MC}}\;\left[1-0.005\;\left(\mathrm{MC}-12\right)\right]$$

## 3. Results and Discussions

Table 3 shows the average values of the MCs achieved in the humidification chamber for each MCt and species, as well as the differences between the achieved MCs and the target MCt.

Except in the case of the values of MCt = 5% for the three species and MCt = 20% for the Western red cedar, the MCs of the specimens within the MCt range ± 1% were obtained. In the case of MCt = 5%, the MC reduction was carried out in the oven, forming a more complex control process.

**Figure 4:**
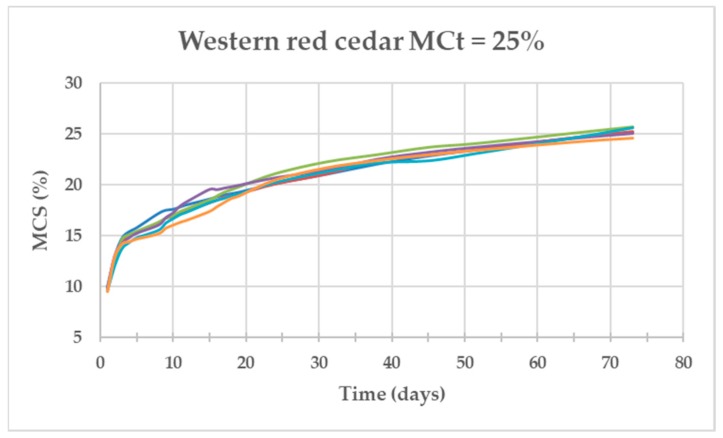
Evolution of the MC of the six Western red cedar specimens as a function of time for MCt = 25%.

**Figure 5:**
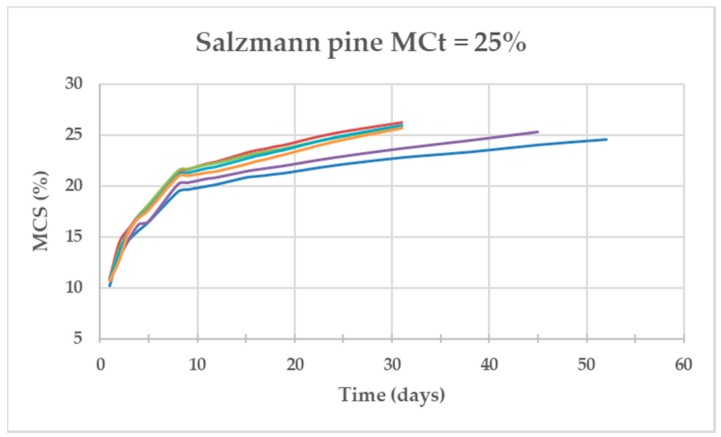
Evolution of the MC of the six Salzmann pine specimens as a function of time for MCt = 25%.

**Figure 6:**
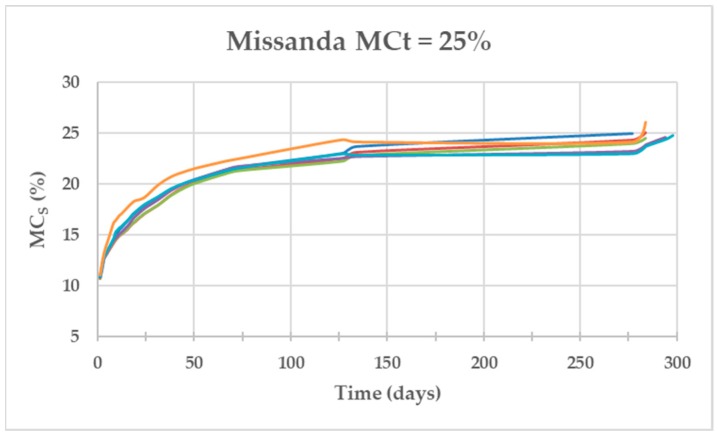
Evolution of the MC of the six Missanda specimens as a function of time for MCt = 25%.

Figure 4, Figure 5 and Figure 6 show the evolution of the MC of the specimens (MCt = 25%) as a function of time for the three species. The Salzmann pine specimens were the fastest in achieving the required MC with an average of 38 days, those of Western red cedar needed twice as many days (73 days) and those of Missanda needed almost eight times more than Salzmann (295 days).

Figure 6 corresponding to Missanda shows that, between day 127 and 274, the MC remained stable around 23% and, between day 274 and 295, a quick MC increase was observed. One possible cause was that the conditioning chamber was placed in the laboratory where there was no control of the outside temperature and during the summer the temperature was very high, which caused the equilibrium moisture of the wood to fall (23% MC). When lowering the temperature of the laboratory (autumn), the equilibrium humidity increased (28% MC), and the specimens increased their moisture content rapidly. For this reason, it is expected that, if the temperature control had been taken, this process would have been cut in half for this species.

Table 4 shows the densities of the three species tested at the different MCt, as well as the wet and dry mass filters filled with chips extracted with the 8 mm drill bit (dr8_w_ and dr8_0_) and with the 7mm drill bit (dr7_w_ and dr7_0_). As in previous research works [21], pine had higher coefficients of variation due to its different percentage of sapwood and heartwood.

### 3.1. Moisture Content Estimation

Table 5 shows the average values of the MC of the specimens (MC_S_) and the chips extracted with the 8 mm drill bit (MC_8_) and with the 7 mm drill bit (MC_7_) for each species and MCt. Since all P-values obtained with the Kolmogorov–Smirnov test are greater than 0.05, it is assumed that the distributions of the different variables (MC_S_, MC_8_ and MC_7_) come from a normal distribution at 95% probability.

Since the P-value (< 0.0001) of the F-test of the ANOVA test is less than 0.05, there is a statistically significant difference between the means of the three variables (MCs, MC_8_, MC_7_) with a 5% level of significance. To determine which means are significantly different from others, multiple range tests were performed. Table 6 shows that MC_8_ and MC_7_ are homogeneous with each other, but both are different from MC_S_. Therefore, the MC of the chips extracted by either of the two prototypes cannot be used as a direct determination of the MC of the specimens.

**Figure 7:**
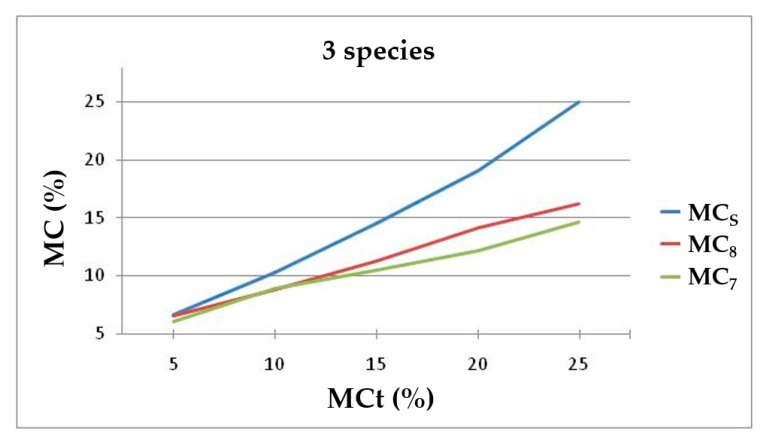
Trend lines of MCs, MC_8_ and MC_7_ (%) as a function of MCt for all three species together.

Figure 7 shows differences increasing between the MC of the extracted chips (MC_8_ and MC_7_) and the MC of the specimens (MC_S_) at a higher MC. The chips extracted with the 7 mm drill lose more moisture than those extracted with the 8 mm drill. However, the data form a homogeneous group.

A model for specimens’ MC estimation based on the mass of the wet and dry filters filled with the drilling residue for each of the drill diameters was developed. Since these variables are not independent of each other, a multiple regression model cannot be made with them. To solve the problem of the models for all three species, an arithmetic transformation of the variables was carried out, replacing dr8_w_ and dr8_0_ with dr8_w_/dr8_0_ and dr7_w_ and dr7_0_ with dr7_w_/dr7_0_ as independent variables, respectively.

The resulting model for estimating the MCs for all three species together for the 8mm drill bit, using dr8_w_/dr8_0_ as an independent variable is shown in Equation (4), where MC_S_ is the MC of the specimen (%); dr8_w_ is the wet mass of the filter filled with drilling residue extracted with the 8 mm drill bit (g), and dr8_0_ is the dry mass of the filter filled with oven-dried drilling residue (g).

(4) $${\mathrm{MC}}_{s}=160.92\times \left(\frac{\mathrm{dr}8_{w}}{\mathrm{dr}8_{0}}\right)-164.16\;R^{2}=86.28\%\;\;\;\;\mathrm{StE}=2.43$$

The standard error of the estimate (StE) indicates that the standard deviation of the residues is 2.43% MC. This error may be significant depending on the estimated MC, from 10% to 50% of the average value in the range from 25% to 5% MC. Kolmogorov–Smirnov Goodness-of-Fit tests were performed for model residuals. Because the smallest P-value (0.287119) from the tests performed is higher than 0.05, the residuals are assumed to come from a normal distribution with 95% confidence. The model also meets the hypotheses of linearity, homoscedasticity and independence of the residues.

In the case of 7 mm drill bit, the resulting model for estimating the MC of the specimen for all three species, using dr7_w_/dr7_0_ as an independent variable, is shown in Equation (5), where MC_S_ is the MC of the specimen (%); dr7_w_ is the wet mass of the filter filled with drilling residue extracted with the 7 mm drill bit (g), and dr7_0_ is the dry mass of the filter filled with oven-dried drilling residue (g).

(5) $${\mathrm{MC}}_{s}=161.66\times \left(\frac{\mathrm{dr}7_{w}}{\mathrm{dr}7_{0}}\right)-163.42\;R^{2}\;=71.39\%\;\mathrm{StE}=3.53$$

The StE of the estimate indicates that the standard deviation of the residues is 3.53% MC. This error may be significant depending on the estimated moisture content, from 14% to 70% of the average value in the range from 25% to 5 % MC. Kolmogorov–Smirnov Goodness-of-Fit Tests were performed for model residuals. Because the smallest P-value (0.0578421) from the tests performed is higher than 0.05, the residuals are assumed to come from a normal distribution with 95% confidence. The model also meets the hypotheses of linearity, homoscedasticity and independence of the residues.

These models allow the estimation of MC in a wide range of densities (from 355 to 978 kg m^−3^), and the model using the 8 mm drill provides a higher R^2^ and lower StE in the deviation of the residues.

### 3.2. Direct Estimation of the Density Adjusted at 12% MC

The estimation of the density and MC of the specimens from the wet and dry mass of drilling residue was possible. Now, it is proposed to go one step further and develop a model to estimate the density at a reference of 12% MC, based on the mass of wet and dry drilling residue. To do this, the density value adjusted to 12% MC must be calculated.

The average values of density of the specimens at the different MCt, as well as the adjusted values at 12% MC according to the European standard EN 384:2016+A1:2018 [50] are shown in Table 7.

**Figure 8:**
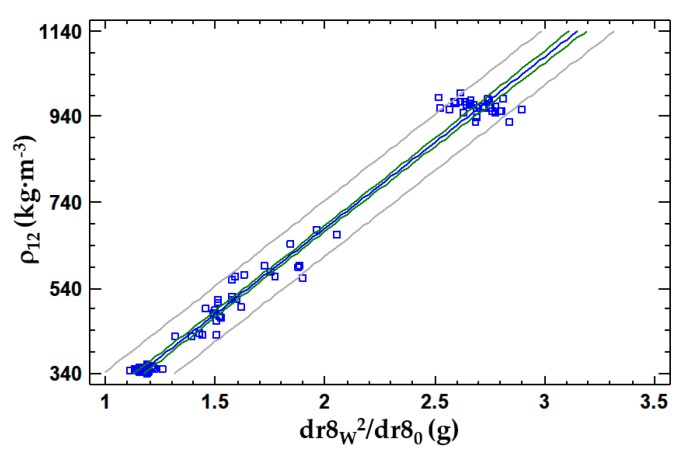
Linear regression model of the density of the specimen adjusted at 12% MC according to dr8_w_ and dr8_0_.

A simple linear regression model was proposed to describe the relationship between the adjusted at 12% MC density of specimens (ρ_12_) and the dr8_w_ and dr8_0_, using (dr8_w_)^2^/dr8_0_ as an independent variable. The resulting model is shown in Equation (6) and Figure 8, where ρ_12_ is the density of the specimen adjusted at 12% MC (kg·m^−3^); dr8_w_ is the wet mass of the filter full of drilling residue extracted with an 8 mm drill bit (g); dr8_0_ is the dry mass of the filter full of drilling residue extracted (g). Kolmogorov–Smirnov Goodness-of-Fit Tests were performed for model residuals. Because the smallest P-value (0.854864) from the tests performed is higher than 0.05, the residuals are assumed to come from a normal distribution with 95% confidence. The model also meets the hypotheses of the linearity, homoscedasticity and independence of the residues.

(6) $$\rho_{12}=399.9\times \frac{\;\mathrm{dr}8_{w}{}^{2}}{\mathrm{dr}8_{0}}-120.1\;R^{2}\;=98.5\%\;\mathrm{StE}=32.6$$

**Figure 9:**
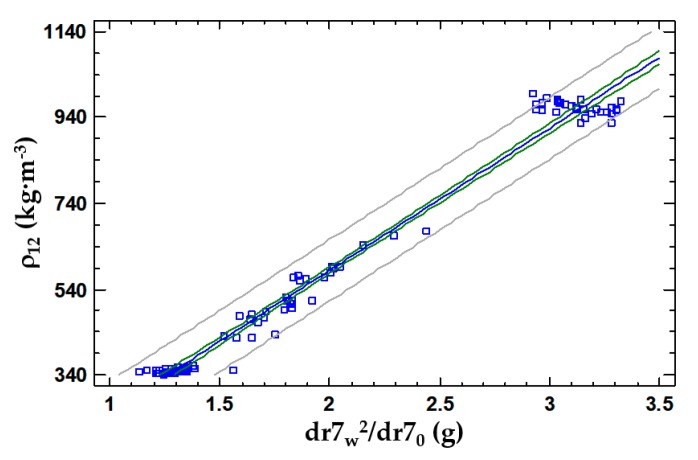
Linear regression model of the density of the specimen adjusted at 12% MC according to dr7_w_ and dr7_0_.

The same statistical analysis was made for the 7 mm drill bit device. A simple linear regression model is proposed to describe the relationship between the adjusted at 12% MC density of specimens (ρ_12_) and the dr7_w_ and dr7_0_, using (dr7_w_)^2^/ dr7_0_ as an independent variable. The resulting model is shown in Equation (7) and Figure 9, where ρ_12_ is the density of the specimen adjusted at 12% MC (kg·m^−3^); dr7_w_ is the wet mass of the filter full of drilling residue extracted with a 7 mm drill bit (g); dr7_0_ is the dry mass of the filter full of drilling residue extracted (g).

Kolmogorov–Smirnov Goodness-of-Fit Tests were performed for model residuals. Because the smallest P-value (0.793982) from the tests performed is higher than 0.05, the residuals are assumed to come from a normal distribution with 95% confidence. The model also meets the hypotheses of linearity, homoscedasticity and independence of the residues.

(7) $$\rho_{12}=330.2\times \frac{\;\mathrm{dr}7_{w}{}^{2}}{\mathrm{dr}7_{0}}-76.2\;R^{2}=98.1\%\;\mathrm{StE}=35.9$$

The R^2^ for both models are very high values. This is due to the great gap between the densities in the three species studied and the low number of specimens required for the wetting process to be viable in a reasonable period of time for the experiment. Both models comply with the hypothesis of the departure and behaviour of their residues. The 8 mm drill model has a slightly higher R^2^ and lower standard error, which makes it a better estimation model. It should be noted that the difference in these values is minimal.

## 4. Conclusions

Chip drill extraction was successfully used for moisture content estimation. The MC of the chips extracted cannot be directly used as specimens’ MC because these are statistically significantly different. This difference is higher, as the MC is higher.

Wood moisture content was estimated by the variable defined by the ratio of dry mass to wet mass of chips extracted. As a result, the determination coefficient for the 8 mm drill bit is 86%, and it is 71 for the 7 mm drill bit.

The regression models used to estimate the density to 12% were therefore calculated using the dry and wet mass of the filters containing the chips extracted. In this case, the determination coefficients rose until 98% for both drill bit models.

The chips drilling extraction methods is an accurate and reliable technique to estimate MC and density using only a measurement in existing structures. Furthermore, when MC is estimated only to adjust density results, models can be used to directly obtain density adjusted to 12% MC.

The proposed models have not been checked in practice yet, but it seems to be a useful tool that provides vital information on the inspection and rehabilitation of existing timber structures.

## Figures and Tables

**Table 1:** Species and specimens’ nominal dimensions.

Common Name	Botanical Name	No. of Pieces	Nominal Length (mm)	Nominal Width (mm)	Nominal Thickness (mm)
Western red cedar	*Thuja plicata* Donn ex D.Don	33	38	90	60
Salzmann pine	*Pinus nigra* Arnold. ssp. *salzmannii* (Dunal) Franco	33	38	90	65
Missanda	*Erythrophleum* sp. Afzel ex G. Don	33	38	100	70

**Table 2:** Example of the process of obtaining moisture content (MCt) for a Western red Cedar specimen of each moisture content group.

Group	MCt (%)	MC_S_ (%)	m_w_ (g)	m_MCt_ (g)	(m_MCt−1_; m_MCt+1_) (g)	Process to Perform	Next Step
1	5	10.04	72.49	69.17	(68.51; 69.83)	Dry	Drying oven
2	10	10.04	71.54	71.51	(70.86; 72.16)	Stabilized	Storage bag
3	15	10.04	72.06	75.31	(74.65; 75.96)	Moisturize	Humidification chamber
4	20	10.04	71.84	78.34	(77.69; 79.00)	Moisturize	Humidification chamber
5	25	10.04	73.48	83.47	(82.80; 84.14)	Moisturize	Humidification chamber

**Table 3:** Average values and coefficients of variation (CV) of MCs of the specimens stabilized to different target moisture contents (MCt).

MCt (%)	Species	Mean MCs (%)	CV (%)	Differences with MCt (%)
5	Western red cedar	7.01	0.78	2.01
	Salzmann pine	6.58	3.30	1.58
	Missanda	6.22	7.43	1.22
10	Western red cedar	9.94	0.39	−0.06
	Salzmann pine	10.65	2.44	0.65
	Missanda	10.30	0.76	0.30
15	Western red cedar	14.36	1.68	−0.64
	Salzmann pine	14.28	3.55	−0.72
	Missanda	14.96	2.32	−0.04
20	Western red cedar	18.41	1.92	−1.59
	Salzmann pine	19.64	3.90	−0.36
	Missanda	19.27	2.70	−0.73
25	Western red cedar	24.56	1.27	−0.44
	Salzmann pine	25.90	9.86	0.90
	Missanda	24.48	2.29	−0.52

**Table 4:** Average values and coefficients of variation of specimens’ density and chips mass by species.

Sp.	MCt (%)	Specimens Density		Chips Mass 8 mm Bit				Chips Mass 7 mm Bit			
				dr8_W_		dr8_0_		dr7_W_		dr7_0_	
		Mean (kg·m^−3^)	CV (%)	Mean (g)	CV (%)	Mean (g)	CV (%)	Mean (g)	CV (%)	Mean (g)	CV (%)
Western red cedar	5	341.20	0.74	1.11	2.47	1.04	2.40	1.25	1.57	1.19	1.50
	10	350.84	1.56	1.11	1.25	1.02	1.18	1.27	2.75	1.17	1.43
	15	353.05	1.64	1.08	1.36	0.99	1.79	1.17	1.68	1.07	2.55
	20	362.03	0.79	1.03	2.08	0.92	2.38	1.11	4.75	1.01	4.35
	25	370.84	1.91	1.05	2.54	0.91	2.81	1.11	2.93	0.99	2.66
	All	355.59	3.18	1.07	3.51	0.98	5.89	1.18	6.48	1.09	7.91
Salzmann pine	5	523.53	13.60	1.46	10.32	1.35	9.81	1.72	10.03	1.61	10.16
	10	554.44	12.13	1.50	10.90	1.37	11.03	1.78	13.40	1.62	13.26
	15	514.65	14.27	1.46	12.02	1.30	11.04	1.68	8.05	1.51	8.15
	20	516.46	16.58	1.39	14.76	1.21	15.22	1.54	15.50	1.37	15.23
	25	556.80	11.43	1.41	11.30	1.21	11.65	1.56	11.54	1.35	11.84
	All	533.17	13.13	1.44	11.41	1.29	12.22	1.65	12.39	1.49	13.50
Missanda	5	946.46	1.51	2.43	1.36	2.30	1.21	2.82	1.60	2.67	1.53
	10	965.77	0.51	2.46	1.92	2.26	1.87	2.75	3.59	2.54	3.56
	15	977.84	0.95	2.45	2.14	2.19	2.29	2.84	2.75	2.56	2.70
	20	983.73	1.25	2.42	2.46	2.09	2.59	2.80	1.92	2.46	1.91
	25	1017.71	1.60	2.30	1.58	1.96	1.47	2.81	1.55	2.41	1.57
	All	978.30	2.70	2.41	2.88	2.17	5.81	2.80	2.46	2.53	4.14
All	5	603.73	43.70	1.67	34.97	1.57	35.52	1.93	35.30	1.82	35.45
	10	623.68	42.61	1.69	35.06	1.55	34.94	1.93	33.49	1.78	33.85
	15	615.18	44.77	1.66	36.16	1.49	35.52	1.90	38.15	1.71	37.83
	20	620.74	44.46	1.61	38.34	1.41	37.20	1.82	41.36	1.61	40.06
	25	648.45	43.50	1.54	34.75	1.32	34.07	1.83	40.80	1.59	39.55
	All	622.36	42.89	1.63	35.22	1.47	35.28	1.88	37.04	1.70	36.80

**Table 5:** Average values of the specimen’s moisture contents (MC_S_), the chips extracted with the 8 mm drill bit (MC_8_) and with the 7 mm drill bit (MC_7_) and their coefficients of variation for each species and MCt.

Sp.	MCt (%)	MC_S_		MC_8_		MC_7_	
		Mean (%)	CV (%)	Mean (%)	CV (%)	Mean (%)	CV (%)
Western red cedar	5	7.01	0.78	6.07	7.60	5.32	7.91
	10	9.94	0.39	7.98	4.80	8.86	42.66
	15	14.36	1.68	9.82	11.79	9.37	33.32
	20	18.41	1.92	11.79	7.84	9.54	9.90
	25	24.56	1.27	14.82	4.83	12.26	4.43
	All	14.86	42.54	10.10	31.36	9.07	33.93
Salzmann pine	5	6.58	3.30	7.97	9.72	7.07	4.80
	10	10.65	2.44	9.51	5.68	9.66	9.47
	15	14.28	3.55	11.95	9.67	11.18	5.71
	20	19.64	3.90	14.84	5.95	13.03	4.12
	25	25.90	9.86	16.93	10.30	15.18	4.13
	All	15.41	45.35	12.24	28.67	11.22	25.76
Missanda	5	6.22	7.43	5.65	6.43	5.69	4.46
	10	10.30	0.76	8.86	6.13	8.26	4.64
	15	14.96	2.32	11.98	1.92	10.79	3.25
	20	19.27	2.70	15.72	4.06	13.88	4.52
	25	24.48	2.29	16.81	8.42	16.37	5.20
	All	15.05	43.61	11.80	36.43	11.00	35.60
All	5	6.60	6.54	6.56	17.77	6.03	13.90
	10	10.30	3.22	8.78	9.05	8.92	24.65
	15	14.53	3.26	11.25	12.20	10.45	18.32
	20	19.11	3.95	14.11	13.45	12.15	16.86
	25	24.98	6.32	16.19	10.01	14.60	12.94
	All	15.10	43.42	11.38	33.08	10.43	32.86

**Table 6:** Multiple Range Tests for MCs, MC8 and MC7. Method: 95.0 percent LSD.

Variable	Mean (%)	Homogeneous Group	
MC_S_	15.10	X	
MC_8_	11.38		X
MC_7_	10.43		X

**Table 7:** Average of the densities of the specimens for the different MCt and adjusted at 12% MC.

Species	MCt (%)	Density at MCt		Density Adjusted at 12% MC	
				EN 384:2016+A1:2018	
		Mean (kg·m^−3^)	CV (%)	Mean (kg·m^−3^)	CV (%)
Western red cedar	5	341.20	0.74	349.72	0.75
	10	350.84	1.56	354.44	1.56
	15	353.05	1.64	348.88	1.57
	20	362.03	0.79	350.42	0.90
	25	370.84	1.91	347.55	1.80
	All	355.59	3.18	350.20	1.45
Salzmann pine	5	523.53	13.60	537.69	13.59
	10	554.44	12.13	558.17	12.10
	15	514.65	14.27	508.64	14.06
	20	516.46	16.58	496.78	16.73
	25	556.80	11.43	518.12	11.62
	All	533.17	13.13	523.88	13.35
Missanda	5	946.46	1.51	973.81	1.48
	10	965.77	0.51	973.99	0.50
	15	977.84	0.95	963.38	0.99
	20	983.73	1.25	947.98	1.31
	25	1017.71	1.60	954.19	1.64
	All	978.30	2.70	962.67	1.60
All	5	603.73	43.70	620.40	43.84
	10	623.68	42.61	628.87	42.59
	15	615.18	44.77	606.97	44.60
	20	620.74	44.46	598.39	44.39
	25	648.45	43.50	606.62	43.69
	All	622.36	42.89	612.25	42.86
